# Bovine Necrotic Vulvovaginitis Associated with *Porphyromonas levii*

**DOI:** 10.3201/eid1003.020592

**Published:** 2004-03

**Authors:** Daniel Elad, Orly Friedgut, Nir Alpert, Yehuda Stram, Dan Lahav, Doron Tiomkin, Miriam Avramson, Kalia Grinberg, Michael Bernstein

**Affiliations:** * Kimron Veterinary Institute, Bet Dagan, Israel; † Hahaklait, Caesarea, Israel; ‡ Veterinary Field Services, Israel

**Keywords:** *Porphyromonas levii*, Bovine Herpesvirus 4, Dairy Cattle, Necrotic Vulvovaginitis

## Abstract

An outbreak of bovine necrotic vulvovaginitis associated with *Porphyromonas levii*, an emerging animal and human pathogen, affected 32 cows on a dairy farm in the northeast of Israel. Five animals had to be culled. This report appears to be the first that associates *P. levii* with bovine necrotic vulvovagnitis.

*Porphyromonas levii* is an emerging pathogen of human and veterinary importance. *P. levii*–like microorganisms were isolated from various human and animal infections (*1**–**5*). Investigation of *P. levii* virulence factors (*6**,**7*) indicated that this microorganism is able to synthesize an anti-IgG_2_ protease (*8*) and to reduce macrophage chemotaxis, phagocytosis, and oxidative burst. These activities were obviated by the presence of anti-*P. levii* serum or immunoglobulin (Ig) G, indicating that acquired immunity might be important in preventing infection (*4*).

In Israel, for the last several years, a tendency has been to combine dairy herds into large holdings, for economic reasons. Thus, large groups of cattle are often moved and introduced into new environments that might substantially differ from those where they originated. During the end of 2000 and the beginning of 2001, outbreaks of bovine necrotic vulvovaginitis (BNVV), lasting about 4 months, were observed in three dairy herds in northeast Israel, several months after the introduction of new stock. Heifers and transferred cows were affected more frequently than multiparous and local cows, respectively.

Towards the end of 2001, two similar outbreaks of BNVV were reported, one of them in a previously unaffected herd. The heifers of this herd were transferred as calves to be raised on another farm and returned before calving. BNVV was not observed on the hosting farm. A third outbreak began in January 2002 on a farm that had been affected during the same season 1 year previously. This outbreak was followed from its onset to its conclusion and is described in this report.

## Outbreak

The herd comprised approximately 550 dairy cows of the Israeli-Holstein breed raised in a zero-grazing management system. Heifers were transferred at the age of 2 months to another farm and returned pregnant 14 months later. All the heifers were kept together in one group. All the cows and heifers were observed at least twice daily by the herd’s personnel and at least twice weekly by the attending veterinarian.

**Figure 1:**
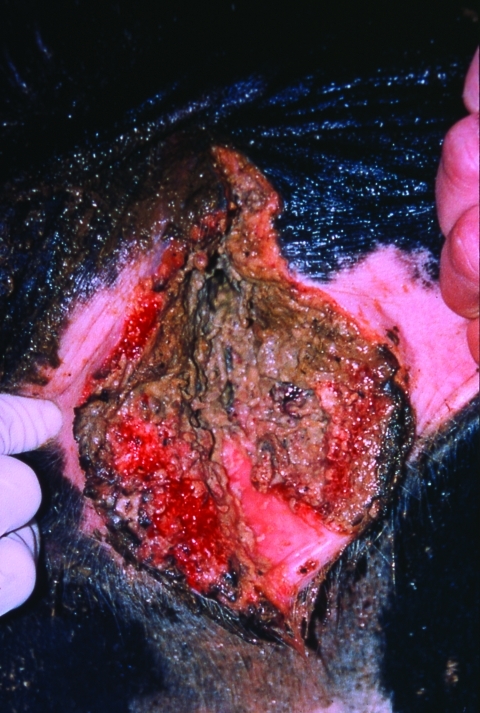
Hemorrhagic vaginal necrosis characteristic of advanced bovine necrotic vulvovaginitis.

Cases of BNVV were observed during January through March 2002. Thirty-nine heifers calved during this period and were included in this study. Clinical signs of BNVV developed in 32 of the 39 heifers. Other age groups and heifers before calving were not affected. Clinical signs appeared during the first week after calving. The prodromic phase of the lesions was characterized primarily by erythema. Subsequently, the lesions progressed to hemorrhagic necrosis (Figure 1). Affected cows were not isolated from the rest of the herd. Treatment consisted of vaginal rinses with potassium permanganate or H_2_O_2_ daily from calving for as long as clinical symptoms persisted (*9*). Local antimicrobial therapy was not attempted since it was ineffective during the outbreaks on the other farms. A penicillin/streptomycin combination was given to 12 cows with temperatures >39.5°C, to provide broad-spectrum protection and prevent sepsis and limit economic losses (metronidazole is the drug of choice to treat infections caused by anaerobic bacteria, but its use is prohibited in Israel). Five of these cows developed metritis and peritonitis and had to be slaughtered, whereas the temperature of the remaining seven cows returned to normal. The systemic treatment had no clinical or bacteriologic effect on the vulvovaginal lesions. No difference between convalescence periods of cows treated with potassium permanganate or H_2_O_2_ was observed. Systemic antimicrobial therapy had no discernable effect on the vulvovaginal lesions. Eighteen cows recovered within 4 weeks after onset of infection; in 9, a more chronic infection, characterized by a mucopurulent vaginal discharge lasting up to 10 weeks, developed.

Laboratory diagnosis was performed on samples taken at various stages of the outbreak. The perineum of each cow was washed and disinfected with 70% alcohol before sampling. Vaginal swabs for bacteriology were taken weekly. The swabs were kept in Amies Transport Medium (Copan, Italy) and processed within 5 hours from sampling. Swabs for mycoplasma and ureaplasma were taken in Mycoplasmal Transport Medium and Hayflick’s Medium, respectively (10). Swabs for virologic examination were placed in Eagle’s medium supplemented with antimicrobials and fetal calf serum and chilled. After vortexing, the medium was used for polymerase chain reaction (PCR) assays and virus isolation. A total of 136 samples from 39 cows were examined bacteriologically. From these, 82 samples from 38 cows were examined virologically as well. Vaginal biopsies were suspended directly in 10% formalin and stained with hematoxylin-eosin in the laboratory.

Bacteriologic examination included aerobic, microaerophilic, and anaerobic cultures, and resulting microorganisms were identified by standard methods (*10*). Samples were examined for *Chlamydia* by direct immunofluorescence (Cellab, Australia) and for *Coxiella burnetti* by the Stamp (*11*) staining method. The only microorganisms cultured consistently from affected cows but not from healthy ones were pigmented, gram-negative, non–spore-forming, anaerobic rods. Autosatellitism was observed in several instances. The number of pigmented colonies was directly related to the severity of the vulvovaginal lesions, and they were not cultured from cows after their recovery.

The pigmented bacteria were weakly saccharolytic; negative for indole, catalase, esculin hydrolysis, α-fucosidase, and α-galactosidase; and positive for β-galactosidase and N-acetyl-β-glucosaminidase positive (as determined by the API rapid ID 32A kit, [bioMérieux, France]). Identification of pigmented isolates as *P. levii* was done according to the Manual of Clinical Microbiology (*12*). Since susceptibility to vancomycin distinguishes *Porphyromonas* spp. from other gram-negative anaerobic rods (*12*) the MIC of the isolates to this antibiotic was determined by the Etest method (AB Biodisk, Sweden) and was found to be 3 μg/mL, indicating susceptibility. Other potentially pathogenic bacteria isolated occasionally included *Arcanobacterium pyogenes*, α-hemolytic streptococci, and several *Enterobacteriaceae*, but they could not be correlated with either the clinical signs or the presence of *P. levii* or bovine herpesvirus 4 (BoHV-4).

For the virologic examination, samples were added to confluent monolayers of Madin-Darby bovine kidney cells, observed daily for cytopathic effect, and passaged every 7 days. Samples were considered negative if no cytopathic effect appeared within 3 weeklong passages. Isolates of BoHV-4 were identified by PCR (L. Koznetzova, et al., unpub. data). BoHV-4 was detected in samples from 27 cows with BNVV and from two cows in which the syndrome did not develop. It was not found in four healthy and five infected cows. Homology with the bB gene of BoHV-4 was >98%. BoHV-1 was isolated from 11 samples, alone or in conjunction with BoHV-4.

Association of the bacteriologic or virologic findings and the clinical status of the cows was assessed statistically by the Mantel-Haenszel test (Statistix, Analytical Sotware, Tallahassee, FL). Data used for the Mantel-Haenszel test are presented in the Table. The test indicated a significant association between cows with BNVV and the presence of *P. levii*, adjusted for BoHV-4 (p = 0.0006). No statistically significant association between BoHV-4, adjusted for *P. levii,* and BNVV was found (p = 0.2359). Previous publications (*13*) also reported difficulties establishing a clear relationship between the presence of BoHV-4 and, among other syndromes, bovine metritis, vaginitis, and abortions.

**Table:** Results of bacteriologic and virologic examinations in outbreak of bovine necrotic vulvovaginitis in Israeli-Holstein dairy cows on a farm in the northeast of Israel^a^

Findings	No. cows in which clinical signs present	No. cows in which clinical signs absent
*P. levii* isolated	32	2
*P. levii* not isolated	0	5
BoHV-4 found	26	2
BoHV-4 not found	5	5

^a^*P. levii*, *Porphyromonas levii*; BoHV-4, bovine herpesvirus 4.

**Figure 2:**
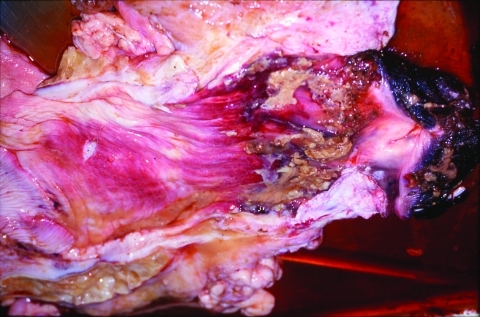
Pathologic lesions of the vagina; note involvement primarily of caudal region.

Uterine biopsies were not taken, as injuring the mucosa in the presence of vaginal lesions could increase the risk for metritis. One cow, diagnosed with metritis and peritonitis, was necropsied. Bacterial colonies were seen in the section of vaginal biopsies, but typical changes of BoHV-4 infection (*13*) were absent. The single necropsied cow showed peritonitis, resulting from rectal rupture, the cause of which could not be determined. Numerous pseudomembranous ulcers, covered with pus, were seen on the vaginal mucosa. The lesions affected the vulva and caudal part of the vagina but not the uterus (Figure 2). Histopathologic examination of the vaginal lesions showed extensive necrosis of the epithelium and severe infiltration with a large number of mostly degenerative neutrophils and foamy macrophages. In the submucosa, vascular proliferation was evident.

## Conclusions

Since *P. levii* was isolated from all BNVV cases and very few healthy cows, it likely caused the lesions. Statistical analysis of data corroborates this assumption. All the cases observed during this outbreak occurred in primiparous cows in a restricted geographic area (within a radius of 20 km) during a limited period of time, indicating that one or more risk factors, alone or in conjunction, predisposed cows to infection. Outbreaks were observed after a large number of cattle were introduced, affecting primarily the animals introduced into the host farm. Transportation and social conflict may have acted as stressors (with stress’ immunosuppressive effects) (*14*) and predisposed the introduced cows to infection, with only social conflict affecting the local cattle. An additional risk factor might have been the age and primiparity of the cows. Calving is a well-known stressor (*15*), especially in primiparous cows (*16*). Moreover, lesions of the genital tract caused by calving tend to be more severe in primiparous cows, thus making them more prone to infection.

After BNVV was described and its putative etiologic agent was identified, several sporadic cases were diagnosed on other farms, indicating that it might be underdiagnosed, and further studies of the syndrome may be warranted.
